# Effect of Pharmacological Agents Administered for Attenuating the Extubation Response on the Quality of Extubation: A Systematic Review

**DOI:** 10.7759/cureus.6427

**Published:** 2019-12-20

**Authors:** Bushra Salim, Saima Rashid, M Asghar Ali, Amir Raza, Fauzia A Khan

**Affiliations:** 1 Anaesthesiology, Aga Khan University, Karachi, PAK

**Keywords:** endotracheal extubation, complications, cough, dexmedetomidine, lidocaine, opioids

## Abstract

Background

Several drugs have been tried to obtund the hemodynamic extubation response but all have variable side effects that may affect the quality of short-term recovery.

Objective

Our primary objective was to evaluate the effect of pharmacological agents, such as dexmedetomidine, local anesthetics, and so on, administered for attenuating the extubation response on the quality of extubation, as judged by the presence or absence of cough, sedation, and laryngospasm/bronchospasm in adult patients who had undergone general anesthesia. A secondary objective was to evaluate the effect of these drugs on other immediate post-extubation complications such as respiratory depression, desaturation, bradycardia, hypotension, and nausea and vomiting (PONV).

Methods

This is a systematic review of (randomized controlled trials)* *RCTs with meta-analysis. The Medical Literature Analysis and Retrieval System Online (MEDLINE), Cumulative Index of Nursing and Allied Health Literature (CINAHL), and Cochrane Central Register of Controlled Trials (CENTRAL) databases were searched for RCTs on the effect of pharmacological agents on both the hemodynamic extubation response as well as the quality of extubation.

Results

Fourteen out of 24 included studies were subjected to a meta-analysis. The risk of cough was less likely in the intervention group as compared to control groups (OR 0.26, 95% CI 0.15 to 0.46, p<0.00001, I^2^=35%). Sedation, hypotension (OR= 10.47; 95% CI: 1.86, 58.80, p=0.008, I^2^=0%), and bradycardia (OR= 6.57; 95% CI: 2.09, 20.64, p=0.001, I^2^=0%) were reported with dexmedetomidine. Only one study reported laryngospasm with dexmedetomidine and two studies with opioids.

Conclusion

Dexmedetomidine 0.4 to 0.5 ug/kg was associated with smooth extubation, minimal coughing, no laryngospasm/ bronchospasm, and with stable hemodynamics, without causing respiratory depression, PONV, and desaturation. However, in higher doses (more than 0.5 ug/kg), it caused bradycardia, hypotension, and sedation. Other pharmacological agents, such as local anesthetics, calcium channel blockers, and opioids, did not attenuate cough associated with extubation.

## Introduction

Tracheal extubation following general anesthesia is associated with hemodynamic changes and airway reflexes [1]. The goals of smooth extubation are to avoid hemodynamic changes, minimize airway stimulation, and prevent straining, coughing, breath-holding, and laryngospasm, as well as to ensure continuous oxygen delivery to the lungs. Patients with cardiovascular and/or neurological diseases, active and passive smokers, and those with chronic airway diseases have a higher incidence of complications as related to extubation [1].

Several drugs have been investigated to obtund the hemodynamic extubation response in vulnerable patients. These are narcotics [2-3], local anesthetics [4], calcium channel blockers [5], alpha agonists, and so on [6-7]. All these pharmacological interventions are associated with certain undesirable side effects [4].

The rationale of this systematic review was to determine the effectiveness of the pharmacological agents administered for attenuating the hemodynamic extubation response with minimal effects on the quality of tracheal extubation.

Objectives

Our primary objective was to evaluate the effect of pharmacological agents administered for attenuating the tracheal extubation response on the quality of extubation as judged by the presence or absence of cough and/or sedation and the presence of laryngospasm/bronchospasm in adult patients undergoing general anesthesia. Our secondary objective was to evaluate the effect of these drugs on other, immediate post-extubation complications such as respiratory depression, desaturation, bradycardia, hypotension, and nausea and vomiting.

## Materials and methods

Design

A systematic review of randomized controlled trials (RCTs) with a meta-analysis.

Data sources

The Medical Literature Analysis and Retrieval System Online (MEDLINE), Cumulative Index of Nursing and Allied Health Literature (CINAHL), and Cochrane Central Register of Controlled Trials (CENTRAL) databases were systematically searched for articles published between January 1, 1990, and December 31, 2015 (26 years).

The search strategy used and the keywords are provided in the appendix.

A bibliography of relevant articles was searched for additional studies and the search was not restricted by language. Authors of identified publications were not contacted for additional information.

Eligibility criteria

Inclusion Criteria

We included RCTs that studied the effect of pharmacological agents on both the hemodynamic extubation response as well as the quality of extubation. RCTs with both placebo and a drug control group, reporting on adult patients (18 years or above), of any race, either gender, and undergoing elective surgery in the operating room were included.

Studies that reported on any of the following primary or secondary outcomes were included.

Primary outcomes: The primary outcome was the quality of extubation. This was assessed by the presence or absence of cough at the time of extubation (graded from 1 to 5) [8], degree of sedation after extubation (Ramsay scale score of 1 and 2 meaning no sedation) [9-10], and the presence of laryngospasm/ bronchospasm at the time of extubation.

Secondary outcomes: The secondary outcome were respiratory depression (respiratory rate less than 10 breaths per minute), bradycardia (heart rate less than 60 beats per minute), hypotension (blood pressure less than 20% from the baseline), nausea and vomiting, desaturation (peripheral capillary oxygen saturation (SpO2) less than 92%) and any other adverse effects of drugs used for the suppression of the hemodynamic extubation response.

Exclusion Criteria

Studies where different doses of routine anesthetic drugs were used, (induction agents, muscle relaxants or inhalation agents) for attenuating the hemodynamic response to extubation were excluded.

Studies of patients undergoing tracheal extubation outside the operating room were also excluded.

Screening and Study Eligibility

All abstracts were independently screened by two reviewers. The selected articles were again reviewed independently by two reviewers. Any disagreement was referred to the third reviewer. The reasons for the exclusion of studies were also noted.

Data extraction and handling

Data were extracted individually by two reviewers on a predesigned data extraction form.

Assessment of Risk of Bias in Individual Studies

The risk of bias assessment was noted appropriately by the authors according to a standard description for each type of bias based on the Cochrane risk of bias tool [11]. Random sequence generation (selection bias), allocation concealment (selection bias), blinding of participants (performance bias), blinding of outcome assessment (detection bias), bias of incomplete outcome data (attrition bias), and selective reporting bias (reporting bias) were assessed. After an independent assessment and then comparison, any conflicts were resolved by a discussion with the third reviewer. The studies were categorized into good quality, fair quality, and poor quality according to the thresholds set for converting the Cochrane risk of bias tool to Agency for Health Care Research and Quality (AHRQ) standards [11].

Statistical analysis 

Meta-analyses were performed using Review Manager, version 5 software (The Cochrane Collaboration, Oxford, UK). The rate of cough, hypotension, bradycardia and nausea/vomiting of the intervention and control groups were tabulated and presented graphically using forest plots. The Mantel-Haenszel (M-H) analysis method with the random-effects model was used to compute the effect size in terms of the odds ratio for dichotomous outcomes. The chi-square (π2) test and I2 were performed to observe variability in the intervention effect that was due to heterogeneity among studies.

## Results

Study selection

**Figure 1:**
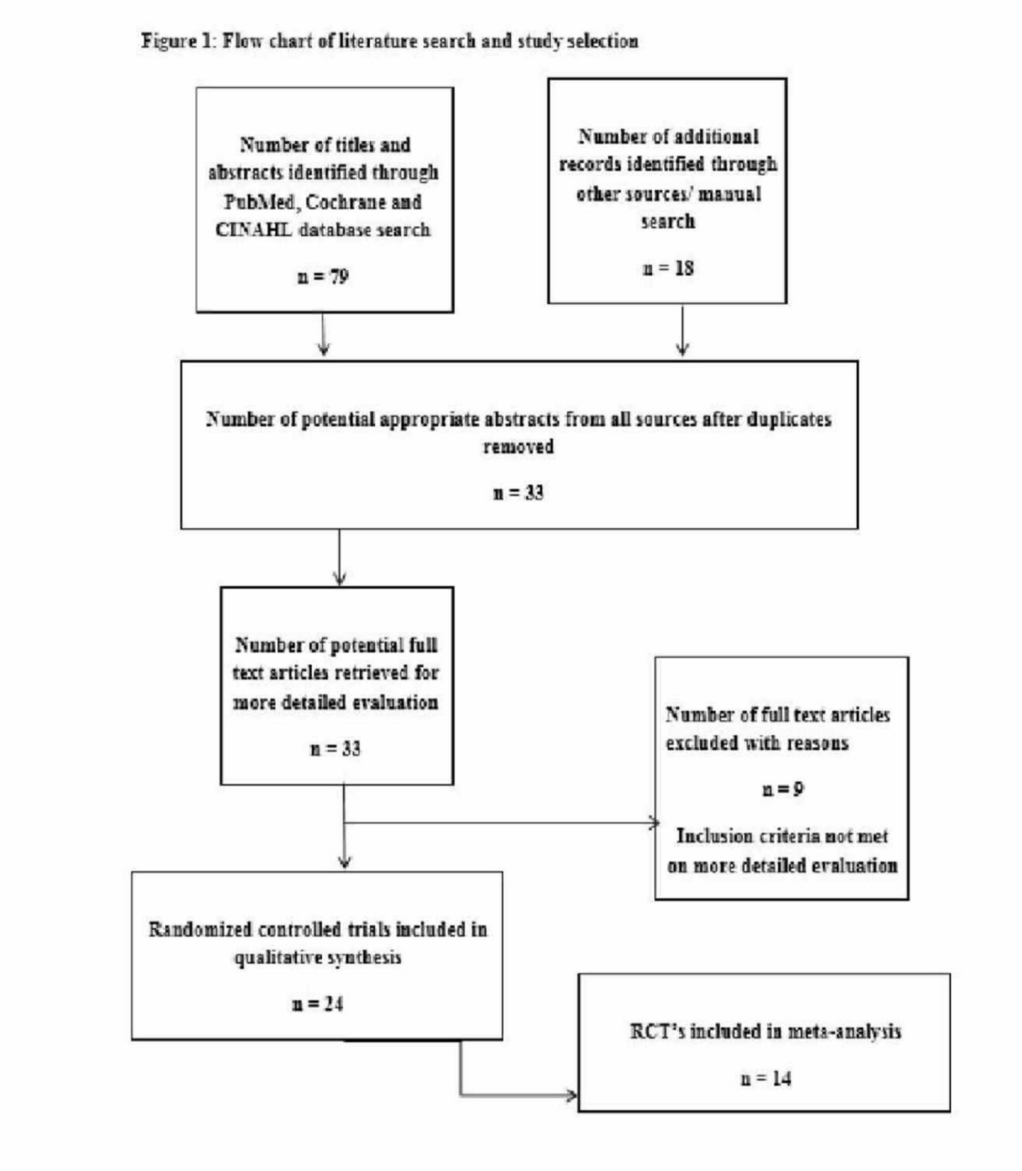
Flow chart of literature search and study selection

Our literature search identified 33 abstracts through both a database and a manual search. After going through the full texts of the abstracts, nine were excluded, as they did not fulfill our inclusion criteria completely, hence, 24 studies were included in the qualitative analysis (Figure 1).

The data of all the study characteristics are shown in Table 1.

**Table 1:** Characteristics of included studies IV: intravenous; TCM: transcricoid membrane; N/S: normal saline; n: group sample size; N: total sample size; min: minutes

Author/ Year	N	Study Groups	Dose	Per Group (n)	Route of Administration	Timing
Nishina 1995 [2]	60	Saline		20	Bolus	At time of peritoneal closure
		Fentanyl	1 ug/kg	20		
		Fentanyl	2 ug/kg	20		
Aksu 2009 [3]	40	Dexmedetomidine	0.5 ug/kg	20	Infusion	Before extubation
		Fentanyl	1 ug/kg	20		
Mistry 2016 [5]	30	Verapamil	0.1mg/kg	15	IV Bolus/Infusion	On return of breathing
		Dexmedetomidine	0.3ug/kg	15		
Kim 2015 [6]	115	Saline	0.1 ml/kg/hr	28(a), 30(b)	Infusion	Drug given after induction
		Dexmedetomidine	0.4 ug/kg/hr	27(a), 30(b)	Infusion	
		Lidocaine	1mg/kg	25	I.V Bolus/Infusion	
		PG.E	0.1/mg/kg	25	Infusion	
		PG.L	0.1/mg/kg	25	Bolus + Infusion	
Xiaochun 2014 [7]	90	Saline		30	IV Bolus	30 minutes after intubation
		Dexmedetomidine	0.4 ug/kg	30	IV Bolus	
		Dexmedetomidine	0.8 ug/kg	30	IV Bolus	
Mikawa 1996 [12]	80	Saline		20	I.V/ Bolus	3 min after reversal
		Diltiazem	0.2 mg/kg	20		
		Verapamil	0.5 mg/kg	20		
		Verapamil	0.1 mg/kg	20		
Nishina 1997 [13]	100	Saline	1mg/kg	25	I.V	2 min before extubation
		Lidocaine	1mg/kg	25	I.V Bolus/Infusion	
		PG.E	0.1/mg/kg	25	Infusion	
		PG.L	0.1/mg/kg	25	Bolus + Infusion	
Jee 2002 [14]	75	Control		25	IV Bolus	3 to 5 min before extubation
		Lidocaine	1 mg/kg 2 %	25	IV Bolus	
		Lidocaine	1 mg/kg 2 %	25	Intra tracheally	
Guler 2005 [15]	60	Dexmedetomidine	0.5mg/kg	30	I.V bolus	5 min before end of surgery
		Saline		30		
Mahoori 2014 [16]	50	Saline		25	Bolus	90 sec prior to extubation
		Remifentanil	0.2 ug/kg	25	Bolus	
Andrzejowski 2002 [17]	40	Saline	5ml	20	Tube cuff	Insertion of first skin clip
		Lidocaine	2% 5ml	20	Tube cuff	
Lee 2014 [18]	142	Saline		71	Infusion	After extubation
		Dexmedetomidine	0.5 ug/kg	71	Infusion	
Shajar 1999 [19]	40	Saline		20	I.V/ Bolus	At time of last suture
		Remifentanil	1 ug/kg	20		
Moustafa 2012 [20]	60	Lidocaine	1.0mg/kg	20	Bolus	5 min before extubation
		Dexmedetomidine	1 mg/kg	20		
		Dexa +Lidocaine	0.1 ug/kg + 1 mg/kg	20		
Nho 2009 [21]	40	Saline		20	Infusion	4 min post extubation	
		Remifentanil		20			
Aouad 2009 [22]	60	Saline		30	Infusion	At the end of the surgery	
		Remifentanil	1/10^th ^dose of infusion	30	Infusion		
Qing Fan 2015 [23]	74	Sevoflurane-Remifentanil	0.03 ug/kg/min	25	Infusion	10 min before extubation	
		Sevoflurane-Dexmedetomide SD5	0.5 ug/kg	24			
		Sevoflurane​​​​​​​-SD7	0.7 ug/kg	25			
Dutta 2016 [24]	45	Saline	10 ml	15	Endotracheally	After last skin suture	
		Lidocaine	1.5 mg/kg	15	Endotracheally		
		Dexmedetomidine	0.3 ug/kg	15	IV		
Turan2008 [25]	40	Saline		20	Bolus over 60 second	5 min before end of procedure	
		Dexmedetomidine	0.5 ug/kg	20	Bolus over 60 second		
Sharma 2014 [26]	60	Saline	10 ml	20	Bolus	Just before extubation	
		Lidocaine	1.5 mg/kg	20	Bolus		
		Dexmedetomidine	0.5 ug/kg	20	Bolus		
Gao 2014 [27]	70	Ropivaciane	20mg	35	TCM	Before intubation	
		Diacine	20mg	35	TCM		
Kothari 2014 [28]	50	Dexmedetomidine	0.5 ug/kg	25	IV bolus	5 minutes before extubation	
		Lignocaine	1.5 mg/kg	25			
Bindu 2013 [29]	50	Saline	100 ml	25	I.V infusion	15 min before extubation	
		Dexmedetomidine	0.75 mcg/kg	25	I.V infusion		
Shruthi 2016 [30]	80	Saline	10 ml	40	Infusion	Beginning of skin closure	
		Dexmedetomidine	0.5 ug/kg	40	Infusion		

Hemodynamic changes

The hemodynamic response was reported as blood pressure (BP) and heart rate (HR) change in all trials but the manner of reporting was different among studies. Nine studies documented a change in systolic blood pressure (SBP), diastolic blood pressure (DBP), and HR [2-3,7,12-17] while 12 studies documented the changes in mean arterial pressure (MAP) and HR only [5-6,18-27]. Three studies documented changes in MAP in addition to SBP, DBP, and HR [28-30]. A saline control group was used in 18 studies [2,6-7,12-19,21-22,24-26,29-30]. In four studies, no placebo was used in the control against the study drug [3,20,27-28]. In seven studies, the authors compared two different drugs or the same drug in different doses [3,5,12,20,23,27-28].

Hypotension was recorded in three studies [15,29-30] while bradycardia was observed in seven studies (see Table 2) [3,5,15,18,26,29-30].

**Table 2:** Attenuation of hemodynamic response in the included studies HR: heart rate; SBP: systolic blood pressure; DBP: diastolic blood pressure; MAP: mean arterial pressure

Study ID Year	Attenuation of Haemodynamic Response	Drug Groups	Comments		
Nishina 1995 [2]	Yes	Fentanyl Saline	HR, SBP, DBP higher in the control group as compared to fentanyl (p<0.05)	
Mikawa 1996 [12]	Yes	Diltiazem, Verapamil, Saline	HR, SBP, DBP. Both drugs attenuated but verapamil 0.1 mg /kg more effective			
Nishina 1997 [13]	Yes	Lidocaine, PGE, PGE, Lidocaine Saline	PGE, Lidocaine combination attenuated SBP, DBP, and HR (p<0.05)	
Shajar 1997 [19]	Yes	Remifentanil Saline	Remi attenuated both MAP, HR in comparison with saline (p<0.01 and 0.05)	
Jee 2002 [14]	Yes	Lidocaine Saline	HR, SBP, DBP were attenuated by Lidocaine sprayed down the ETT immediately after extubation only	
Andrzejowski 2002 [17]	No difference	Lidocaine Saline	No difference between the groups (p>0.05)
Guler 2005 [15]	Yes	Dex Saline	SAP and DAP were significantly lower in the dex group compared to saline (p<0.05). Episode of bradycardia in 1 and hypotension in 3 patients in the dex group	
Turan 2008 [25]	Yes	Dex Saline	HR and MAP were significantly higher in control as compared to the dex group (p<0.01)	
Aouad 2009 [22]	Yes	Remifentanil Saline	HR and MAP increased in control as compared to remi (p<0.05)	
Nho 2009 [21]	Yes	Remifentanil Saline	HR and MAP were significantly increased in the control group as compared to remi (HR p=0.001 and MAP p=0.002)	
Aksu 2009 [3]	Yes	Dex Fentanyl	HR, SBP, DBP were significantly increased by in fentanyl group as compared to dex (HR p=0.003and SBP p=0.037)	
Moustafa 2012 [20]	Yes	Lidocaine Dex Dex plus lidocaine	Dex+lidocaine combination attenuated HR, MAP, RPP in comparison to the two drugs alone (p<0.05)	
Bindhu 2013 [29]	Yes	Dex Saline	HR, SBP, DBP, and MAP significantly higher in control (p<0.05). Bradycardia and hypotension reported with dex	
Mahoori 2014 [16]	Yes	Dex Saline	HR, SBP, DBP were significantly increased in control (p<0.05)	
Xiachun^7^ 2014 [7]	Yes	Dex Saline	Dexmedetomidine 0.8 ug/kg more effectively attenuated HR, SBP, and DBP	
Sharma 2014 [26]	Yes	Dex Lidocaine Saline	Dexmedetomidine more effective than lignocaine in attenuating HR (p=0.01), MAP. One patient had bradycardia in the dex group	
Lee 2014 [18]	Yes	Dex Saline	HR, MAP were attenuated in the dex group as compared to control. One patient had bradycardia in the dex group	
Kothari 2014 [28]	Yes	Dex Lidocaine	HR, SBP, DBP, MAP were below baseline in the dex group as compared to the lido group (p<0.05)	
Gao 2014 [27]	Yes	Ropivacaine Diacine	HR, MAP Ropivaciane more effective than diacine ( p<0.05)	
Fan 2015 [23]	Yes	Remifentanil Dex	HR, MAP. Dexmedetomidine more effective than remifentanil (p<0.05)	
Kim^6^ 2015 [6]	Yes	Dex Saline	HR was lower in the dex group (p<0.05), no difference in MAP	
Mistry 2016 [5]	Yes	Verapamil Dex	HR, MAP were higher in the verapamil group than in the dex but statistically insignificant	
Shruthi 2016c[30]	Yes	Dex Saline	HR, SBP, DBP, MAP were lower in the dex group but significantly increased in the control (p<0.001)	
Dutta^24^ 2016 [24]	Yes	Lidocaine Dex Saline	HR, MAP. Dexmedetomidine better effect than lignocaine spray (p<0.05)	

Surrogate measures used for the quality of extubation and the immediate post-extubation complications

The following outcome measures were used for assessing the quality of extubation and the immediate post-extubation complications. The primary outcome measures were cough, sedation, and laryngospasm/bronchospasm. The secondary outcome measures seen were hypotension, bradycardia, and immediate postoperative nausea and vomiting. The outcome measures are summarized in Table 3.

**Table 3:** Primary and secondary outcomes reported in the included studies

Author/Year	Study Groups		Per Group (n)	Primary Outcome (Event/n)				Secondary Outcome (Event/n)				
				Cough	Sedation	Laryngospasm		Hypotension	Desaturation	Bradycardia	Nausea/ vomiting	Respiratory depression
Nishina 1995 [2]	Saline		20	20/20	Zero	Zero		Zero	Zero	Zero	16/20	Zero
	Fentanyl		20	19/20	Zero	Zero		Zero	Zero	Zero	19/20	Zero
	Fentanyl		20	17/20	Zero	Zero		Zero	Zero	Zero	19/20	Zero
Mikawa 1996 [12]	Saline		20	3/20	NR	Zero		Zero	NR	Zero	NR	NR
	Diltiazem		20	3/20	NR	Zero		Zero	NR	Zero	NR	NR
	Verapamil		20	3/20	NR	Zero		Zero	NR	Zero	NR	NR
	Verapamil		20	3/20	NR	Zero		Zero	NR	Zero	NR	NR
Nishina 1997 [13]	Saline		25	25/25	NR	Zero		Zero	NR	Zero	NR	NR
	Lidocaine		25	13/25	NR	Zero		Zero	NR	Zero	NR	NR
	PG.E		25	25/25	NR	Zero		Zero	NR	Zero	NR	NR
	PG.L		25	14/25	NR	Zero		Zero	NR	Zero	NR	NR
Shajar 1999 [19]	Saline		20	11/20	10/20	NR		zero	NR	Zero	1	Zero
	Remifentanil		20	9/20	3/20	NR		zero	NR	Zero	2	Zero
Jee 2002 [14]	Saline			14/25	NR	Zero		NR	NR	NR	NR	NR
	Lidocaine		25	10/25	NR	Zero		NR	NR	NR	NR	NR
	Lidocaine		25	11/25	NR	Zero		NR	NR	Non	NR	NR
Andrzejowski 2002 [17]	Saline		20	Zero	NR	NR		Zero	NR	Zero	NR	NR
	Lidocaine		20	Zero	NR	NR		Zero	NR	Zero	NR	NR
Guler 2005 [15]	Dexmedetomidine		30	3	NR	Zero		3	Zero	1	NR	Zero
	Saline		30	8	NR	Zero		Zero	Zero	Zero	NR	Zero
Turan 2008 [25]	Saline		20	4/20	NR	Zero		Zero	Zero	Zero	Zero	Zero
	Dexmedetomidine		20	0/20	NR	Zero		Zero	Zero	Zero	Zero	Zero
Aouad 2009 [22]	Saline		30	0/30	NR	Zero		Zero	Zero	Zero	NR	Zero
	Remifentanil		30	2/30	NR	Zero		Zero	Zero	Zero	NR	Zero
Nho 2009 [21]	Saline		20	8/20	NR	NR		Zero	Zero	Zero	3/20	Zero
	Remifentanil		20	0/20	NR	NR		Zero	Zero	Zero	0/20	Zero
Aksu 2009 [3]	Dexmedetomidine		20	1 (5%)	1	Zero		Zero	Zero	2/20	2/20	NR
	Fentanyl		20	4 (20%)	2	1		Zero	Zero	2/20	3/20	NR
Moustafa 2012 [20]	Lidocaine		20	5	NR	NR		Zero	Zero	Zero	NR	NR
	Dexmedetomidine		20	14	NR	NR		NR	Zero	NR	NR	NR
	Dexmedetomidine +Lidocaine		20	5	NR	NR		NR	Zero	NR	NR	NR
Bindu 2013 [29]	Saline		25	21/25	5/25	Zero		0/25	Zero	2/25	2/25	Zero
	Dexmedetomidine		25	4/25	21/25	Zero		2/25	Zero	13/25	1/25	Zero
Mahoori 2014 [16]	Saline		25	11/25	NR	1		Zero	Zero	Zero	NR	NR
	Remifentanil		25	6/25	NR	Zero		Zero	Zero	Zero	NR	NR
Xiaochun 2014 [7]	Saline		30	Zero	Zero	NR		Zero	Zero	NR	Zero	NR
	Dexmedetomidine		30	Zero	Zero	NR		Zero	NR	NR	Zero	NR
	Dexmedetomidine		30	Zero	Zero	NR		Zero	NR	NR	Zero	NR
Sharma 2014 [26]	Saline		20	2/20	Zero	Zero		Zero	Zero	0/20	NR	NR
	Lidocaine		20	0/20	Zero	Zero		Zero	Zero	0/20	NR	NR
	Dexmedetomidine		20	0/20	Zero	Zero		Zero	Zero	1/20	NR	NR
Lee 2014 [18]	Saline		71	14/71	3	Zero		Zero	Zero	Zero	Zero	Zero
	Dexmedetomidine		70	5/70	3	Zero		Zero	Zero	1/70	Zero	Zero
Kothari 2014 [28]	Dexmedetomidine		25	Zero	18/25	Zero		Zero	Zero	Zero	NR	Zero
	Lignocaine		25	5	Zero	Zero		Zero	Zero	Zero	NR	Zero
Gao 2014 [27]	Ropivaciane		35	0/35	NR	NR		Zero	Zero	Zero	2/35	Zero
	Diacine		35	4/35	NR	NR		Zero	Zero	Zero	3/35	Zero
Qing Fan 2015 [23]	Sevoflurane-Remifentanil		25	Zero	Zero	Zero		NR	Zero	NR	12/25	NR
	Sevoflurane-Dexmedetomide SD5		24	Zero	Zero	1 (4.2)		Zero	1 (4.2)	NR	4/25	NR
	Sevoflurane-SD7		25	Zero	Zero	Zero		Zero	Zero	NR	4/25	NR
Kim 2015 [6]	Saline		28(a), 30(b)	NR	1(a) 6(b)	NR		Zero	NR	Zero	11/28 (a) 3/30 (b)	NR
	Dexmedetomidine		27(a), 30(b)	NR	13(a) 11(b)	NR		Zero	NR	Zero	9/27 (a) 2/30 (b)	NR
Mistry 2016 [5]	Verapamil		15	Zero	Zero	Zero		Zero	NR	Zero	NR	NR
	Dexmedetomidine		15	Zero	Zero	Zero		Zero	NR	1/15	NR	NR
Shruthi 2016 [30]	Saline		40	12/40	Zero	Zero		0/40	Zero	0/40	NR	NR
	Dexmedetomidine		40	2/40	Zero	Zero		9/40	Zero	2/40	NR	NR
Dutta 2016 [24]	Saline		15	Zero	Zero	Zero		Zero	NR	Zero	NR	NR
	Lidocaine		15	Zero	Zero	Zero		Zero	NR	Zero	NR	NR
	Dexmedetomidine		15	Zero	Zero	Zero		Zero	NR	Zero	NR	NR

Cough

**Figure 2:**
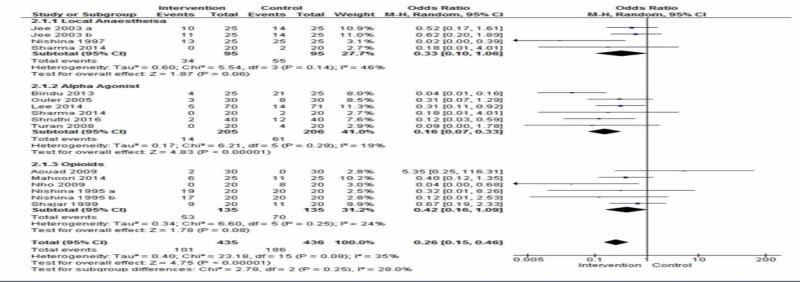
Comparison of incidence of cough between interventions vs. placebo

Cough was observed in 13 placebo-controlled studies [2,13-16,18-19,21-22,25-26,29-30], and all these studies were included in the meta-analysis. Overall, cough developed in 23.2% of patients in the intervention group and 42.6% in the control group. The risk of cough was less likely in the intervention group as compared to the control group (OR 0.26, 95% CI 0.15 to 0.46, p=0.00001, I2=35%) (Figure 2).

The odds ratio was calculated in the following subgroups.

Local Anesthetics Versus Placebo

Three studies compared lidocaine with placebo and were subjected to a meta-analysis [13-14,26]. There were 95 patients in each group, 34 developed a cough in the intervention group and 55 in the control group. The odds ratio was found to be 0.33 (95% CI 0.10 - 1.06) p=0.06) I2 =46%.

Alpha Agonist Versus Placebo

Six studies compared the alpha agonist with the placebo [15,18,25-26,29-30]. The incidence of cough was significantly reduced with alpha agonists. The odds ratio was 0.16 (95% CI: 0.07, 0.33) p< 0.00001) I2= 19%.

Opioids Versus Placebo

In five studies, the authors compared opioids with the placebo [2,16,19,21-22]. There was no statistical significance in the incidence of cough [OR=0.42 95%CI: 0.16, 1.09; p=0.08, I2=24].

Sedation

Sedation was reported in 13 studies using the Ramsay scale [2-3,5-7,18-19,23-24,26,28-30]. These studies compared dexmedetomidine with remifentanil, verapamil, fentanyl, and lidocaine.

Alpha Agonists

Dexmedetomidine in different doses was compared to saline in seven studies [6-7,15,18,25,29-30]. The doses used were 0.4 ug/kg [6-7,18], 0.5 ug/kg [15,25,30], 0.75 ug/kg [29], and 0.8 ug/kg [7]. All the authors reported significantly higher sedation in the patient groups who were administered dexmedetomidine. Dexmedetomidine 0.1 ug/kg resulted in a higher degree of sedation as compared to verapamil 0.3 ug/kg [5], but patients who received verapamil were anxious, agitated, and restless. The results were equivocal in studies that compared dexmedetomidine with lidocaine [24,26]. Dexmedetomidine in a dose of 0.3 and 0.5 ug/kg as compared to lidocaine did not show a significant difference in sedation [24,26]. Two studies compared dexmedetomidine with opioids [3,23]. Dexmedetomidine 0.5 ug/kg was compared with fentanyl 1 ug/kg. One patient in the dexmedetomidine group and two in the fentanyl group were not arousable [3]. Remifentanil 0.03 ug/kg/min was compared with dexmedetomidine 0.5 ug/kg and 0.7 ug/kg [23]. Time to awakening was comparable in all the groups p=0.24.

Opioids

Two studies compared opioids with the placebo [2,19]. Remifentanil 1 ug/kg was compared with saline. Only three patients were sedated in the remifentanil group as compared to saline where 10 patients had sedation, p=0.056 [19]. Two doses of fentanyl 1 ug/kg and 2 ug/kg were compared with saline and none of the patients were moderately or severely sedated in any group [2].

Laryngospasm/ bronchospasm

Laryngospasm/bronchospasm was reported in three studies [3,16,23]. One study looked at the effect of dexmedetomidine 0.5 ug/kg and fentanyl 1 ug/kg before extubation and reported one episode of laryngospasm in the fentanyl group [3]. Another study compared the effect of remifentanil 0.2 ug/kg with saline and reported one episode of laryngospasm in the saline group [16]. Two different doses of dexmedetomidine 0.5 and 0.7 ug/kg were compared with remifentanil 0.03 ug/kg/min in another study, resulting in one episode of laryngospasm in the 0.5 ug/kg dexmedetomidine group [23].

Hypotension

**Figure 3:**
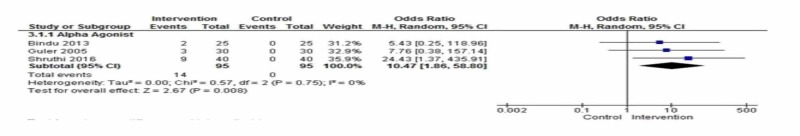
Comparison of the incidence of hypotension between interventions vs. placebo

Hypotension was observed in three studies using alpha agonists [15,29-30]. Fourteen out of 95 patients had hypotension in the intervention group as compared to none in the control group. The odds ratio was 10.47 (CI: 1.86-58.80) with a p-value of 0.008, I2=0% (Figure 3).

Bradycardia

**Figure 4:**
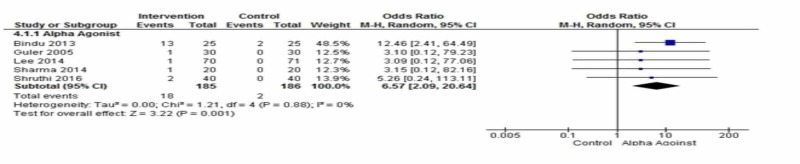
Comparison of the incidence of bradycardia between interventions vs. placebo

Five placebo-controlled studies using dexmedetomidine reported on bradycardia at extubation [15,18,26,29-,30]. All reported bradycardia with dexmedetomidine. Eighteen events of bradycardia occurred in the intervention group as compared to two in the control group. The risk of bradycardia was about seven times more likely in the intervention group as compared to the control group [OR= 6.57; 95% CI: 2.09, 20.64, p=0.001, I2=0%] (Figure 4).

Nausea and vomiting

**Figure 5:**
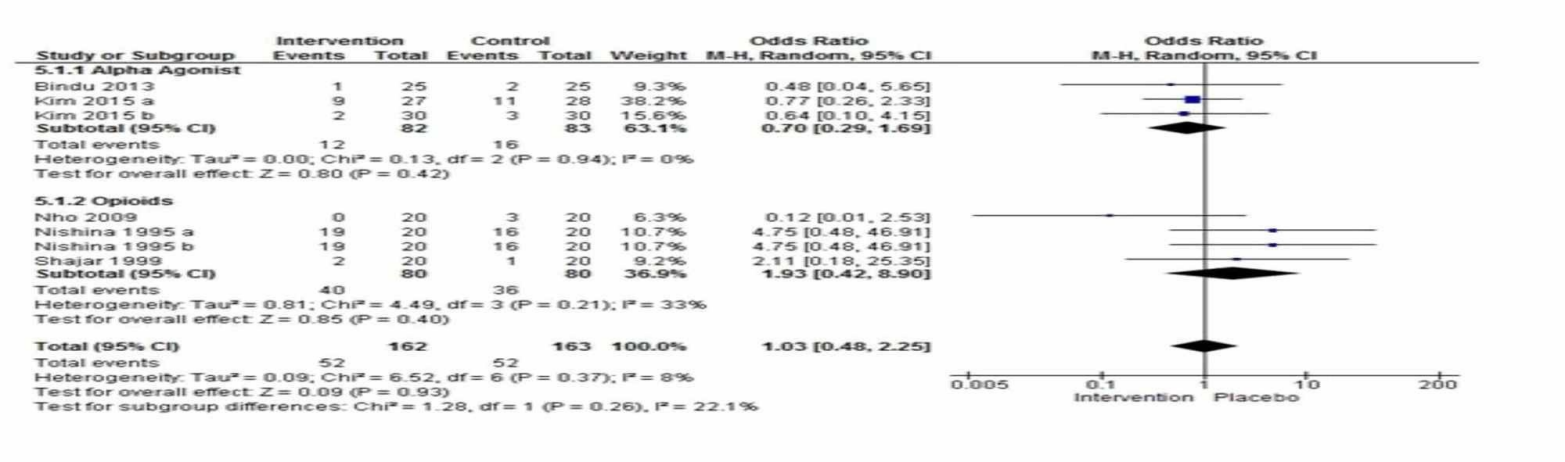
Comparison of the Incidence of Nausea or Vomiting Between Interventions vs. Placebo

Five studies reported nausea and vomiting in the immediate postoperative period [2,6,19,21,29]. The combined effect was not statistically significant between groups [OR= 1.03; 95% CI: 0.48, 2.25, p=0.37, I2=8%] (Figure 5).

Subgroup Analysis for Alpha Agonist and Opioid with Control

Nausea and vomiting were observed and reported in two studies using alpha agonists [3,6] and three studies with opioids [2,19,21].

In the subgroup analysis, the effect was not statistically significant between groups [OR= 0.70; 95% CI: 0.29, 1.68, p=0.42, I2=0%] and [OR= 1.93; 95% CI: 0.42, 8.90; p=0.40, I2=33%].

Studies Not Subjected to the Meta-Analysis

Descriptive results

Studies with Local Anesthetics

Ropivacaine 1% was compared with diacine 1% via a transcricoid membrane injection. In the ropivacaine group, 91.9% (95%CI = 85.2-98.7%) patients did not experience any cough versus 46% (95% CI=34.4-59.2%) patients in the diacine group (P<0.05) [27].

The efficacy of 2% lidocaine administered through the tracheal tube in attenuating the extubation response in patients who were beta blocked with propranolol 1 mg/kg was compared with placebo, resulting in no difference between lidocaine and placebo in the degree of cough (p-value 0.4) [17].

Studies with Prostaglandins

Intravenous lidocaine 1 mg /kg, prostaglandin E 0.1 ug/kg, and a combination of lidocaine and prostaglandin E in the same dose were compared with placebo. Cough was reported in 52% of patients treated with lidocaine alone, 56% with lidocaine prostaglandin E combination while in all patients in prostaglandin E group and placebo [13].

Studies with Alpha Agonists

No patient experienced cough with 0.8 ug/kg dexmedetomidine as compared to 3.3% of patients treated with 0.4 ug/kg dexmedetomidine. This study was in Chinese, and we were not able to get it translated into English; hence, the information presented here is taken from the abstract [7].

Three studies compared dexmedetomidine with lidocaine [24,26,28], the effect of intravenous dexmedetomidine 0.1 ug/ kg was compared with lidocaine 1 mg/kg or their combination in the same dose [20]. Twenty-five percent of patients in the dexmedetomidine group and 5% in both the lidocaine and lidocaine with dexmedetomidine groups developed a severe cough.

Dexmedetomidine 0.3 ug/kg was compared with lidocaine 1.5 mg/kg. The number of patients with no cough was 86.6% in the dexmedetomidine group compared to 60% in the lidocaine group (P=0.0087) [23].

Dexmedetomidine 0.5 ug/kg was compared with lidocaine 1.5 mg/kg. Five patients (20%) had a cough during extubation in the lidocaine group as compared to none in the dexmedetomidine group (p=<0.05) [28].

Dexmedetomidine 0.3 ug/kg, when compared with verapamil 0.1 mg/kg, [5] resulted in 12 (80%) patients in the dexmedetomidine group with no cough while 9 (60 %) in the verapamil group had minimal coughing (P<0.0029).

Two studies compared dexmedetomidine with remifentanil and fentanyl [3,23]. Fan et al. compared two different doses of dexmedetomidine, 0.5 and 0.7 ug/kg, with remifentanil 0.03 ug/kg/min. Only two patients had moderate cough in the remifentanil group, four had moderate, and two had severe cough in the dexmedetomidine 0.5 ug/kg group while none had moderate to severe cough in the dexmedetomidine 0.7 ug/kg group. One patient had laryngospasm in the dexmedetomidine 0.5 group [23]. Aksu et al. studied the effect of dexmedetomidine 0.5 ug/kg and fentanyl 1 ug/kg. No patient had severe cough in the dexmedetomidine group while four had in the fentanyl group. Only one patient (5%) had moderate cough in the dexmedetomidine group in contrast to four (20%) in the fentanyl group (p= 0.003). One patient developed laryngospasm in the fentanyl group [3].

Studies with Narcotics

Nho et al. studied the effect of remifentanil infusion maintained at a target organ concentration of 1.5 ng/ml during emergence. Coughing was less frequent in the remifentanil group than in the control group. They did not give the numbers of patients who experienced cough neither the grade of cough [21].

Studies with Calcium Channel Blockers

Mikawa et al. studied the effect of two different doses of verapamil 0.05 ug/kg and 0.1 ug/kg with diltiazem 0.2 ug/kg and saline. They reported that all patients coughed with the extubation quality scores (median 3, range 2-5) being the same in all the four groups. No patient developed laryngospasm, hypotension, and bradycardia [12].

Risk of bias across studies

The quality of each study was assessed using the Cochrane risk of bias tool for RCTs [27]. This information is given in Table 4.

**Table 4:** Quality assessment of selected studies

	Risk of Bias Assessment

Study ID	Year	Random Sequence Generation	Allocation Concealment	Blinding of Participants and Personnel	Blinding of Outcome Assessment	Incomplete Outcome Data	Selective Outcome Reporting	Quality of Studies
Nishina [2]	1995	unclear	low	low	low	low	low	fair
Mikawa [12]	1996	low	low	low	low	low	low	good
Nishina [13]	1997	unclear	unclear	low	unclear	high	low	poor
Shajar [19]	1999	low	low	low	low	low	low	good
Jee [14]	2002	high	unclear	low	low	low	low	fair
Andrzejowski [17]	2002	unclear	low	low	low	high	low	poor
Guler [15]	2005	unclear	low	low	low	low	low	fair
Turan [25]	2008	unclear	low	low	low	low	low	fair
Aouad [22]	2009	low	low	low	low	low	low	good
Nho [21]	2009	low	low	low	low	low	low	good
Aksu [3]	2009	unclear	low	low	low	low	low	fair
Moustafa [20]	2012	unclear	unclear	low	low	low	Low	fair
Bindu [29]	2013	low	unclear	low	low	low	low	fair
Mahoori [16]	2014	low	unclear	low	low	unclear	low	poor
Zhoo Xiaochun [7]	2014	low	low	low	low	low	low	good
Sharma [26]	2014	Low	low	low	low	low	low	good
Lee [18]	2014	low	low	low	low	low	low	good
Kothari [28]	2014	Low	low	low	low	low	low	good
Gao [27]	2014	low	unclear	low	low	high	low	poor
Qing Fan [23]	2015	low	low	low	low	low	low	good
Kim [6]	2015	low	unclear	low	unclear	low	Low	fair
Mistry [5]	2016	low	low	low	low	low	low	good
Shruthi [30]	2016	low	low	low	low	low	low	good
Dutta [24]	2016	unclear	low	low	low	low	low	fair

## Discussion

The main findings of this review are that at tracheal extubation, dexmedetomidine significantly reduced the incidence of cough but caused hypotension and bradycardia. Local anesthetics and opioids did not cause hypotension and bradycardia at extubation but their effect on cough was equivocal. Nausea and vomiting were observed with opioids, but this was not statistically significant in comparison to saline. Patients who received dexmedetomidine had a higher Ramsay score in recovery when compared to local anesthetics while the results of opioids on sedation were equivocal.

Tracheal extubation is associated with cardiovascular as well as respiratory complications. Hemodynamic complications, such as hypertension may lead to an increase in intraocular and intracranial pressure, tachycardia, and dysrhythmias [12,28]. This can be hazardous in high-risk patients who have hypertension, coronary artery, and /or cerebrovascular disease due to an increase in myocardial oxygen demand, which can lead to further myocardial ischemia and infarction, pulmonary edema, and cerebrovascular hemorrhage [4,6]. Various drugs like beta-blockers, calcium channel blockers, vasodilators, lidocaine, and opioids have been used to attenuate this reflex sympathetic stimulation to extubation, with equivocal results and undesirable side effects like sedation, hypotension, bradycardia, nausea, and vomiting [26,29]. An ideal agent is the one that keeps blood pressure and heart rate stable and has no undesirable side effects. Hemodynamic response was attenuated significantly by all drugs used in all included studies.

Sedation, respiratory depression, agitation, and nausea and vomiting are not desirable during and after extubation. Excessive sedation can lead to respiratory depression and increases morbidity and length of stay in PACU [29]. Similarly, agitation in the postoperative period can be very unpleasant for the patient and can lead to hemodynamic compromise. The aim is to have a calm patient with stable hemodynamics in the recovery room.

Extubation can stimulate unwanted airway responses due to laryngeal and tracheal irritation leading to cough, laryngospasm, and bronchospasm. These airway and circulatory responses on extubation can lead to surgical bleeding, cardiovascular instability, and respiratory compromise [1]. The incidence of post-extubation coughing reported in different studies was between 76% and 96% [1,4,6]. Dexmedetomidine 0.5 ug/kg showed a significant reduction in the incidence of cough after intraocular [15], intracranial [17], and spinal surgeries [24], hence improving the quality of extubation when compared to placebo. It also decreased the need for postoperative analgesia without increasing the duration of stay in recovery [21]. It may cause bradycardia and hypotension in a dose-dependent manner but without other side effects. The dose of dexmedetomidine most commonly used in studies was 0.5 ug/kg but favorable results were seen with doses as low as 0.3 ug/kg [5]. Doses higher than 0.5 ug/kg resulted in higher sedation scores when compared to placebo [7].

Lidocaine alone, given intravenously or intratracheally, failed to produce a favorable outcome on the quality of extubation [24,28]. Combination with other drugs, such as prostaglandin E1 and dexmedetomidine, gave better results. Intravenous lidocaine 1 mg/kg, when used in combination with prostaglandin E1, resulted in good-quality extubation with minimal cough or strain [13]. Laryngotracheal instillation with 2% lidocaine did not produce any difference in the degree of coughing [17]. Only one study reported the use of 2% lidocaine 1 mg/kg spray down the endotracheal tube, which attenuated the airway circulatory reflexes when compared to lidocaine given intravenously in the same dose [14]. Dexmedetomidine and lidocaine in combination when administered intravenously resulted in a favorable quality of extubation when compared with dexmedetomidine 0.1 ug/kg alone [20].

Calcium channel blocker was not found to be effective in the attenuation of cough reflex irrespective of dose and drug used [12]. Short-acting opioids like remifentanil and fentanyl have been used for the suppression of cough reflex, with remifentanil having more favorable effects. Remifentanil infusion resulted in suppressing the cough reflex better than the placebo [21]. Remifentanil infusion has also been effectively used to blunt the cough reflexes after thyroidectomies and nasal surgeries [21-22]. When used in patients undergoing abdominal surgery, remifentanil had no significant difference compared to placebo [16]. This variation can be due to the difference in the type of surgery as well as the use of bolus versus infusion. Fentanyl in a 1 ug/kg dose failed to suppress the cough reflex when compared with 0.5 ug/kg dexmedetomidine [3]. When given in a dose of 2 ug/kg, fentanyl resulted in a lesser incidence of cough compared to 1 ug/kg but that was not statistically significant [2]. Nausea and vomiting were not significantly increased with any of the drugs used in the included studies. The majority of the included studies in this review were of good or fair quality with a low risk of bias. Only three studies had one or more criteria for a high risk of bias.

This review has some limitations. First, not all studies were placebo-controlled. There was heterogeneity among the studies (I2 for cough = 60%). Another limitation was that the population included in most studies was the American Society of Anesthesiologists (ASA) I and II. Only three studies included ASA III patients and only one mentioned the associated co-morbidity present in the patients. The results, therefore, may not be extrapolated to patients with co-morbidity who are those actually at risk of having complications. Further work needs to be done with different doses of dexmedetomidine to recommend a dose attenuating the cough reflex but resulting in stable hemodynamics and a calm patient.

## Conclusions

This meta-analysis results show that dexmedetomidine 0.4-0.5 ug/kg is associated with good-quality smooth extubation, minimal coughing, no laryngospasm/ bronchospasm, and a calm patient, with stable hemodynamics, without causing respiratory depression, nausea and vomiting, and desaturation. However, in higher doses of more than 0.5 ug/kg, it can cause bradycardia, hypotension, and sedation. More studies are needed to find out the ideal dose to be used for the attenuation of extubation response without causing any untoward circulatory depression. Other pharmacological agents, such as local anesthetics, opioids, and calcium channel blockers, did not attenuate cough.

## Appendices

PubMed search

(extubation) OR (tracheal extubation) OR (airway extubation) OR (endotracheal extubation) OR (intratracheal extubation)) AND ((beta blocking drug*) OR (esmolol) OR (labetalol) OR (metoprolol) OR (propranolol) OR (local anesthetic) OR (lidocaine) OR (lignocaine) OR (xylocaine) OR (alpha 2 adrenergic receptor agonists) OR (dexmedetomidine) OR (clonidine) OR (calcium channel blockers) OR (calcium channel antagonist) OR (nicardipine) OR (diltiazem) OR (verapamil) OR (magnesium sulphate)) AND ((cough) OR (dyspnea) OR (apnea) OR (bronchospasm) OR (bronchial spasm) OR (bronchial hyper reactivity) OR (laryngospasm) OR (laryngismus) OR (vocal cord dysfunction) AND (breath holding) OR (propofol) OR (airway obstruction) OR (hypoventilation) OR (hypoxia) OR (hypoxemia) OR (respiratory depression))) AND (Randomized Controlled Trial [ptyp] AND ("1990/01/01"[PDAT]: "2016/12/31"[PDAT]) AND Humans [Mesh] AND adult [Mesh]) (77 items)

CINAHL search

Extubation, airway extubation, Esmolol or labetalol or metoprolol, or propranolol, or lidaocaine, or lignocaine, or xylocaine, or dexmedetomidine, or clonidine, or calcium channel antagonist or nicardipine, or diltiazem, or verapamil, or magnesium sulphate, or dyspnea, or bronchospasm, or laryngospasm or vocal cord dysfunction, or propofol or hypoventilation or hypoxemia, or respiratory depression.

Cochrane database search

Extubation, airway extubation, Esmolol or labetalol or metoprolol, or propranolol, or lidocaine, or lignocaine, or xylocaine, or dexmedetomidine, or clonidine, or calcium channel antagonist or nicardipine, or diltiazem, or verapamil, or magnesium sulfate, or dyspnea, or bronchospasm, or laryngospasm or vocal cord dysfunction, or propofol or hypoventilation or hypoxemia, or respiratory depression.

Cough

1=no coughing

2=smooth extubation, minimal coughing

3=moderate coughing

4=severe coughing

5=poor extubation, very uncomfortable (laryngospasm and coughing 10 times)

1 and 2 mean no coughing.

3, 4, and 5 are yes.

Sedation score

Ramsay Scale

1=anxious or agitated and restless or both

2=cooperative, oriented and tranquil

3=drowsy but responds to commands

4=asleep and brisk response to light glabella tap or loud auditory stimulus

5=asleep, sluggish response to light glabella tap or loud auditory stimulus

6=asleep and unarguable

1 and 2 mean no sedation.

3, 4, 5, and 6 mean yes.

Laryngospasm/ bronchospasm

1=no spasm

2=spasm present

Respiratory depression: respiratory rate of less than 10 breaths per minute

Bradycardia: heart rate of less than 60 b per minute

Hypotension: blood pressure of less than 20 % of baseline

Nausea/vomiting

1=no nausea / vomiting

2=nausea and vomiting present

Desaturation=oxygen saturation of less than 92% immediately after extubation.

Thresholds for converting the Cochrane risk of bias tool to AHRQ Standards (good, fair, and poor)

Good quality: All criteria met (i.e. low for each domain)

Fair quality: One criterion not met (i.e. high risk of bias for one domain) or two criteria unclear, and the assessment that this was unlikely to have biased the outcome, and there is no known important limitation that could invalidate the results

Poor quality: One criterion not met (i.e. high risk of bias for one domain) or two criteria unclear, and the assessment that this was likely to have biased the outcome, and there are important limitations that could invalidate the results

Poor quality: Two or more criteria listed as high or unclear risk of bias
